# Effect of CRP and Kinetics of CRP in Prognosis of Nasopharyngeal Carcinoma

**DOI:** 10.3389/fonc.2019.00089

**Published:** 2019-02-21

**Authors:** Ruiwan Chen, Yu Zhou, Yujie Yuan, Qun Zhang, Shasha He, Yong Chen, Yufeng Ren

**Affiliations:** ^1^Department of Radiation Oncology, The First Affiliated Hospital, Sun Yat-sen University, Guangzhou, China; ^2^Department of General Surgery, Guangdong General Hospital, Guangzhou, China; ^3^Center of Gastroinestinal Surgery, The First Affiliated Hospital, Sun Yat-sen University, Guangzhou, China

**Keywords:** C-reactive protein, nasopharyngeal carcinoma, radiotherapy, kinetics, prognosis

## Abstract

Baseline C-reactive protein (CRP) has been determined as a prognostic factor in nasopharyngeal carcinoma (NPC). This study was designed to further evaluate the impact of CRP kinetics on NPC patients. Thousand three hundred and seventy eight NPC patients from February 2001 to June 2011 were retrospectively reviewed. CRP were measured at beginning, middle, and the end of the treatment. The endpoints were overall survival (OS) and distant metastasis free survival (DMFS). Patients were divided into three groups according to baseline CRP and CRP kinetics: (1) continuously normal group: patients whose baseline CRP normal and never elevated, (2) ever-elevated group: patients whose CRP ever elevated regardless time points, (3) continuously elevated group: patients whose baseline CRP elevated and never normalized. Baseline CRP, CRP after treatment, and CRP kinetics were correlated with TNM stage, T stage, and N stage. Univariate and multivariate analysis identified that elevated baseline CRP and CRP after treatment had significant association with worse survival than normal CRP. Oppositely, elevated CRP during treatment was not associated with survival. Patients with continuously elevated CRP significantly had poor OS and DMFS (HR:2.610, 95%CI: 1.592–4.279, *p* < 0.001; HR:2.816, 95%CI: 1.486–5.302, *p* = 0.001, respectively). In multivariate analysis, CRP kinetics assessment is an independent prognostic factor for OS and DFMS in NPC patients (HR:2.512, 95%CI: 1.452–4.346, *p* = 0.001; HR:3.389, 95%CI: 1.734–6.625, *p* = 0.001, respectively). In conclusion, elevated CRP at baseline and after treatment are predictive factors of poor prognosis for NPC. The study of CRP kinetics shows that continuously elevated CRP during treatment might indicate an unfavorable prognosis for NPC.

## Introduction

Nasopharyngeal carcinoma (NPC) is prevalent in Southern China and Southeast Asia, with a peak incidence of 50 cases per 1,00,000 individuals (1, 2). Radiotherapy with or without chemotherapy is the primary treatment for NPC patients (3). With the rapid development of radiotherapy technology and chemotherapy regimens, the overall 5-year survival rate of NPC is over 75% (4, 5). However, a mysterious heterogeneity remains in clinical outcomes of NPC with the same TNM staging system (6). Recently, numerous evidence has shown that specific biomarkers such as serum lactate dehydrogenase levels (7) and plasma Epstein-Barr virus (EBV) DNA (8) can successfully distinguish prognosis of NPC patients. However, plasma EBV DNA testing has not been routinely applied in clinical practice. It is urgent to explore additional biomarkers that are simple to use and complementary to TNM staging system (9).

Many non-specific inflammatory markers have been detected and proven to play important roles in predicting tumor progression and prognosis (10–12). C-reactive protein (CRP) is one of the most representative markers in the acute phase of systemic inflammatory response (13, 14). It has been reported with increased levels of CRP was relevant to poor prognosis in colorectal cancer (15), hepatocellular carcinoma (16), and esophageal carcinoma (17). Similarly in NPC patients, baseline levels of CRP and CRP/albumin ratio have been both proposed as a potentially useful biomarker (18–21). Besides, dynamic change in CRP levels, known as CRP kinetics, has been reported as a prognostic marker of long-term survival for metastatic NPC patients treated with palliative chemotherapy (22). To our knowledge, the prognostic value of CRP kinetics in non-metastatic NPC remains obscured.

We herein conducted a large-scale retrospective study in a single center and aimed to investigate the role of CRP and CRP kinetics in predicting prognosis of non-metastatic NPC patients treated with radical radiotherapy.

## Materials and Methods

### Patients

From February 2001 to June 2011, 1,378 consecutive patients who had confirmed diagnosis of non-metastatic NPC and received radical radiotherapy in Sun Yat-Sen University Cancer Center were enrolled for initial screening. The selection criteria included: (1) good performance status (KPS ≥ 80); (2) normal renal, cardiac and liver function; (3) at least one CRP record during treatment; and (4) complete follow-up data. This study was approved by the Institutional Review Board of our hospital, with written informed consent exempted from all subjects due to the retrospective study design.

### Treatments

Before any treatment, patients would receive a routine staging work-up, which included a review of patient history, physical examination, magnetic resonance imaging (MRI) scan of nasopharynx and neck, chest radiography, abdominal sonography, and a whole-body bone scan or whole-body fluorodeoxyglucose positron emission tomography-computed tomography (FDG PET/CT). Afterwards, they were staged based on the 8th edition of the International Union against Cancer/American Joint Committee on Cancer (UICC/AJCC) system. Radiotherapy with or without concurrent chemotherapy was followed, with detailed techniques summarized as our previously report.

Serum levels of CRP were measured from peripheral venous blood samples. Baseline CRP was defined as CRP level before any anti-tumor treatment, with CRP during treatment and after treatment defined as values during any cycle of chemotherapy or radiotherapy and in the end of radical radiotherapy, respectively. The cut-off value of CRP was set at 10 mg/L according to previous studies (15, 16, 23). Accordingly, all subjects were intentionally divided into three groups according to baseline CRP and CRP kinetics: (1) non-elevated group: patients whose baseline CRP < 10 mg/L and not elevated after the treatment, (2) ever-elevated group: patients whose baseline CRP or CRP after treatment ever elevated, (3)non-normalized group: patients whose baseline CRP and CRP after treatment were both >10 mg/L.

After treatment, patients were scheduled for regular follow-up visits, who were routinely asked every 3 months during the first 2 years, every 6 months till the 5th year, and then yearly thereafter. Evaluations during each out-patient visit included physical examination, hematology, and biochemistry profiles, MRI scan, chest radiography, abdominal sonography, and a whole-body bone scan.

### Study Outcomes

The primary endpoints were the overall survival (OS) and distant metastasis-free survival (DMFS) after the radical treatment. OS and DMFS were calculated from the first day of treatment to death and the first distant metastasis, respectively.

### Statistical Analyses

All data was stored and analyzed using the statistical package SPSS for Windows (Vers. 22.0, SPSS Inc., Chicago, IL). Pearson's Chi-squared test was used to analyze correlations between different CRP levels and the patients' clinicopathological features. The actuarial survival rates were estimated by the Kaplan-Meier method and survival curves were compared with the log-rank test. Univariate and multivariate analyses were performed using the Cox proportional hazards model. Two-tailed *p*-value < 0.05 were considered statistically significant.

## Results

### Clinical Outcomes

Within the study period, a total of 1,378 non-metastatic NPC patients were included for the final analysis. The median age was 45 years (12–90 years), with 1016 (73.7%) males, and 362 (26.3%) females. The demographic and baseline characteristics of included patients are summarized in Table 1. Briefly, all patients were treated with radiotherapy, including 773 patients with 2-dimensional radiotherapy, 37 patients with 3-dimensional conformal radiation therapy (3D-CRT), and 568 patients with Intensity Modulated Radiation Therapy (IMRT). Additionally, 81.3% (974/1,198) patients received platinum-based chemotherapy, including neoadjuvant, concurrent, or adjuvant chemotherapy for advanced-stage disease. Until the last date of follow-up, the median follow-up period was 52 months (range, 2–106 months), with 150/1,378 (10.8%) and 102/1,378 (7.4%) patients suffering distant metastasis and cancer-related death, respectively. The 5-year OS and DMFS rates were 89.1 and 92.7%, respectively.

**Table 1:** Patient characteristic.

**Characteristics**	***N***	**Baseline CRP, mg/l**			**CRP during treatment, mg/l**			**CRP after treatment, mg/l**			**CRP change**			
		**elevated**	**normal**	***p***	**elevated**	**normal**	***p***	**elevated**	**normal**	***p***	**Non-elevated**	**Ever-elevated**	**Non-normalized**	***p***
**AGE**														
≤ 45y	699	57 (8.2%)	608 (87.0%)	0.676	71 (10.2%)	272 (38.9%)	0.632	23 (3.3%)	222 (31.8%)	0.178	185 (26.5%)	33 (4.7%)	5 (0.7%)	0.542
>45y	679	52 (7.6%)	603 (88.9%)		85 (12.5%)	248 (36.5%)		27 (4.0%)	174 (25.6%)		145 (21.3%)	34 (5.0%)	3 (0.4%)	
**SEX**														
Male	1016	81 (8.0%)	888 (87.4%)	0.824	128 (12.6%)	375 (36.9%)	**0.013**	39 (3.8%)	299 (29.4%)	0.698	247 (24.3%)	50 (4.9%)	7 (0.7%)	0.713
Female	362	28 (7.7%)	323 (89.2%)		28 (7.7%)	145 (40.1%)		11 (3.0%)	97 (26.8%)		83 (22.9%)	17 (4.7%)	1 (0.3%)	
**PATHOLOGY**														
WHO I+II	90	3 (3.3%)	82 (91.1%)	0.102	7 (7.8%)	35 (38.9%)	0.309	0 (0%)	31 (34.4%)	0.040	26 (28.9%)	0 (0%)	0 (0%)	**0.044**
WHO III	1288	106 (8.2%)	1129 (87.7%)		149 (11.6%)	485 (37.7%)		50 (3.9%)	365 (28.3%)		304 (23.6%)	66 (5.1%)	8 (0.6%)	
**BMI**														
< 24	773	67 (8.7%)	674 (87.2%)	0.157	84 (10.9%)	305 (39.5%)	0.235	28 (3.6%)	207 (26.8%)	0.626	174 (22.5%)	38 (4.9%)	5 (0.6%)	0.607
≥24	525	34 (6.5%)	466 (88.8%)		70 (13.3%)	204 (38.9%)		20 (3.8%)	172 (32.7%)		143 (27.2%)	24 (4.6%)	3 (0.6%)	
**FAMILY HISTORY**														
Yes	380	29 (7.6%)	336 (88.4%)	0.812	43 (11.3%)	151 (39.7%)	0.610	0 (0%)	111 (29.2%)	**0.002**	93 (24.5%)	25 (6.6%)	2 (0.5%)	0.236
No	928	74 (8.0%)	812 (87.5%)		105 (11.3%)	332 (35.8%)		23 (2.5%)	251 (27.0%)		207 (22.3%)	34 (3.7%)	4 (0.4%)	
**SMOKING**														
Yes	521	50 (9.6%)	449 (86.2%)	0.062	62 (11.9%)	184 (35.3%)	0.353	22 (4.2%)	136 (26.1%)	0.187	112 (21.5%)	28 (5.4%)	5 (1.0%)	0.142
No	851	58 (6.8%)	758 (89.1%)		94 (11.0%)	332 (39.0%)		28 (3.3%)	258 (30.3%)		216 (25.4%)	39 (4.6%)	3 (0.4%)	
**DM**														
Yes	30	5 (16.7%)	24 (80%)	0.077	4 (13.3%)	12 (40%)	0.859	2 (6.7%)	8 (26.7%)	0.377	6 (20%)	3 (10%)	0 (0%)	0.373
No	1343	104 (7.7%)	1182 (88.0%)		152 (11.3%)	506 (37.7%)		48 (3.6%)	386 (28.7%)		322 (24.0%)	64 (4.8%)	8 (0.6%)	
**HYPERTENSION**														
Yes	75	7 (9.3%)	68 (90.7%)	0.732	10 (13.3%)	28 (37.3%)	0.630	0 (0%)	28 (37.3%)	0.051	25 (33.3%)	3 (4%)	0 (0%)	0.482
No	1300	102 (7.8%)	1140 (87.7%)		146 (11.2%)	491 (37.8%)		50 (3.8%)	366 (28.2%)		303 (23.3%)	64 (4.9%)	8 (0.6%)	
**CHRONIC HBV INFECTION**														
Yes	236	17 (7.2%)	213 (90.3%)	0.618	36 (15.3%)	91 (38.6%)	0.123	6 (2.5%)	55 (23.6%)	0.674	47 (20.0%)	9 (3.8%)	1 (0.4%)	0.961
No	1070	86 (8.0%)	939 (87.9%)		115 (10.7%)	410 (38.3%)		43 (4.0%)	325 (30.4%)		272 (25.4%)	57 (5.3%)	7 (0.7%)	
**TNM STAGE**														
I+II	306	10 (3.3%)	286 (93.5%)	**0.001**	22 (7.2%)	97 (31.7%)	0.191	5 (1.6%)	79 (25.8%)	0.090	69 (22.5%)	7 (2.2%)	0 (0%)	0.053
III+IV	1080	99 (9.2%)	925 (85.6%)		134 (12.4%)	423 (39.2%)		45 (4.2%)	317 (29.3%)		261 (24.2%)	60 (5.6%)	8 (0.7%)	
**T STAGE**														
1+2	529	25 (4.7%)	479 (90.5%)	**0.001**	52 (9.8%)	193 (36.5%)	0.389	14 (2.6%)	161 (30.4%)	0.084	138 (26.1%)	15 (2.8%)	4 (0.8%)	**0.010**
3+4	849	84 (9.9%)	732 (86.2%)		104 (12.2%)	327 (38.5%)		36 (4.2%)	235 (27.7%)		192 (22.6%)	52 (6.1%)	4 (0.5%)	
**N STAGE**														
0+1	752	48 (6.4%)	677 (90.0%)	**0.017**	68 (9.0%)	268 (35.6%)	0.082	17 (2.3%)	194 (25.8%)	**0.045**	162 (21.5%)	27 (3.6%)	0 (0%)	**0.016**
2+3	626	61 (9.7%)	534 (85.3%)		88 (14.1%)	252 (40.3%)		33 (5.3%)	202 (32.3%)		168 (26.8%)	37 (5.9%)	8 (1.3%)	
**RADIOTHERAPY**														
2D+3D-CRT	810	62 (7.7%)	717 (88.5%)	0.629	91 (11.2%)	279 (34.4%)	0.303	22 (2.7%)	190 (23.5%)		157 (19.4%)	30 (3.7%)	2 (0.2%)	0.424
IMRT	568	47 (8.3%)	493 (86.8%)		65 (11.4%)	241 (42.4%)		28 (4.9%)	206 (36.3%)		173 (30.5%)	37 (6.5%)	6 (1.0%)	
**CHEMOTHERAPY**														
Yes	974	89 (9.1%)	839 (86.1%)	0.019	132 (13.6%)	421 (43.2%)	0.054	41 (4.2%)	319 (32.8%)	0.532	265 (27.2%)	55 (5.6%)	8 (0.8%)	0.289
No	224	10 (4.5%)	207 (92.4%)		9 (4.0%)	58 (25.9%)		5 (2.2%)	53 (23.7%)		47 (21.0%)	6 (2.7%)	0 (0%)	

*p < 0.05 was considered statistically significant*.

### CRP and CRP Kinetics

**Figure 1:**
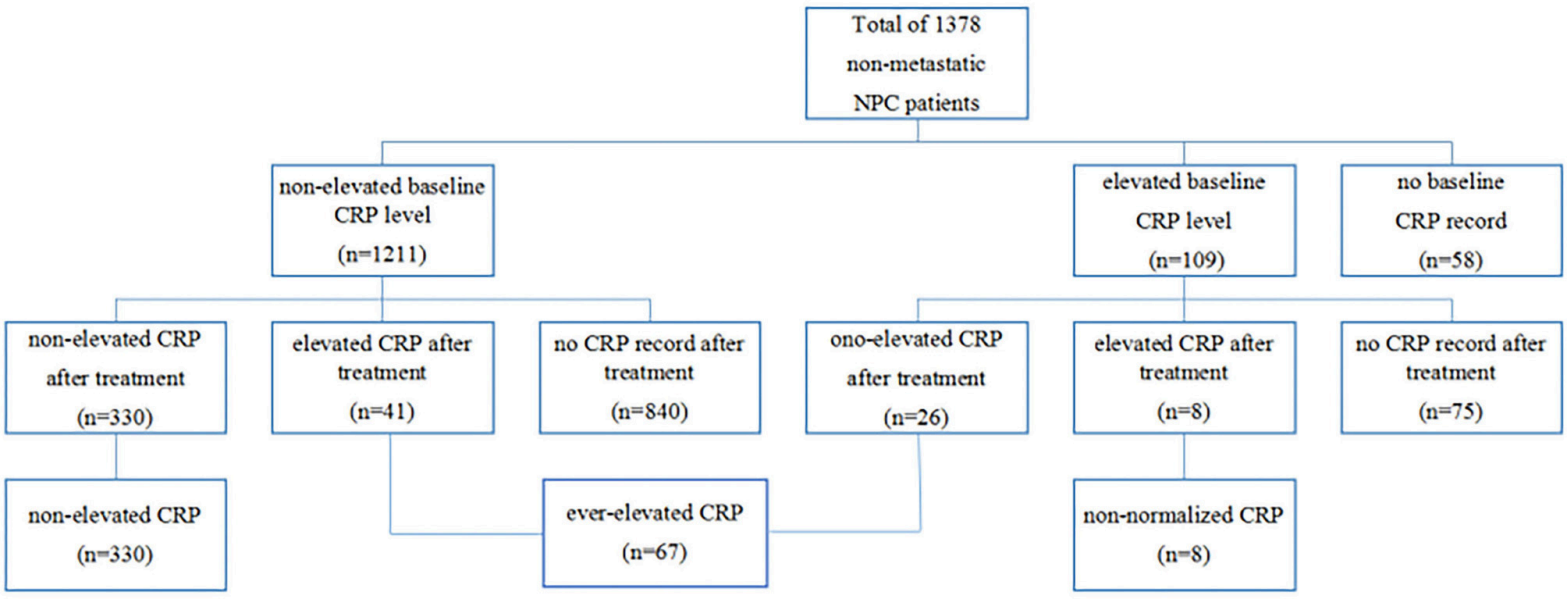
Flow chart of the different patients group according to CRP kinetics.

In the entire cohort, 109 (8.3%) patients had elevated CRP levels at baseline measurement, 1,211 (87.9%) patients had normal CRP levels, and 58 (4.2%) patients had missing values. After treatment, 330 of 1,211 patients with previous normal baseline CRP still had non-elevated CRP, but 8 of 109 patients with previous elevated CRP still had elevated CRP. On the basis of our grouping method, 330, 67 and 8 patients were assigned into non-elevated group, ever-elevated group, and non-normalized group, respectively (Figure 1).

### Association of CRP Kinetics With Tumor Stage

The chi-squared test results showed that our data was relatively balanced.

Patients with T3-4, N2-3, and Stage III-IVa disease had significantly higher baseline levels of CRP than those with T1-2, N0-1, and Stage I-II disease (*p* = 0.001, 0.017, and 0.001, respectively). Similarly, there is a trend that patients with worse T, N, and total stage had relatively higher CRP after treatment (*p* = 0.090, 0.084, and 0.045, respectively) and CRP kinetics (*p* = 0.053, 0.01, and 0.016, respectively) in comparison with early stage patients. However, elevated CRP during treatment was not associated with advanced tumor stage (*p* = 0.191, 0.389, and 0.082, respectively).

### CRP Levels Impacting On Survival

**Figure 2:**
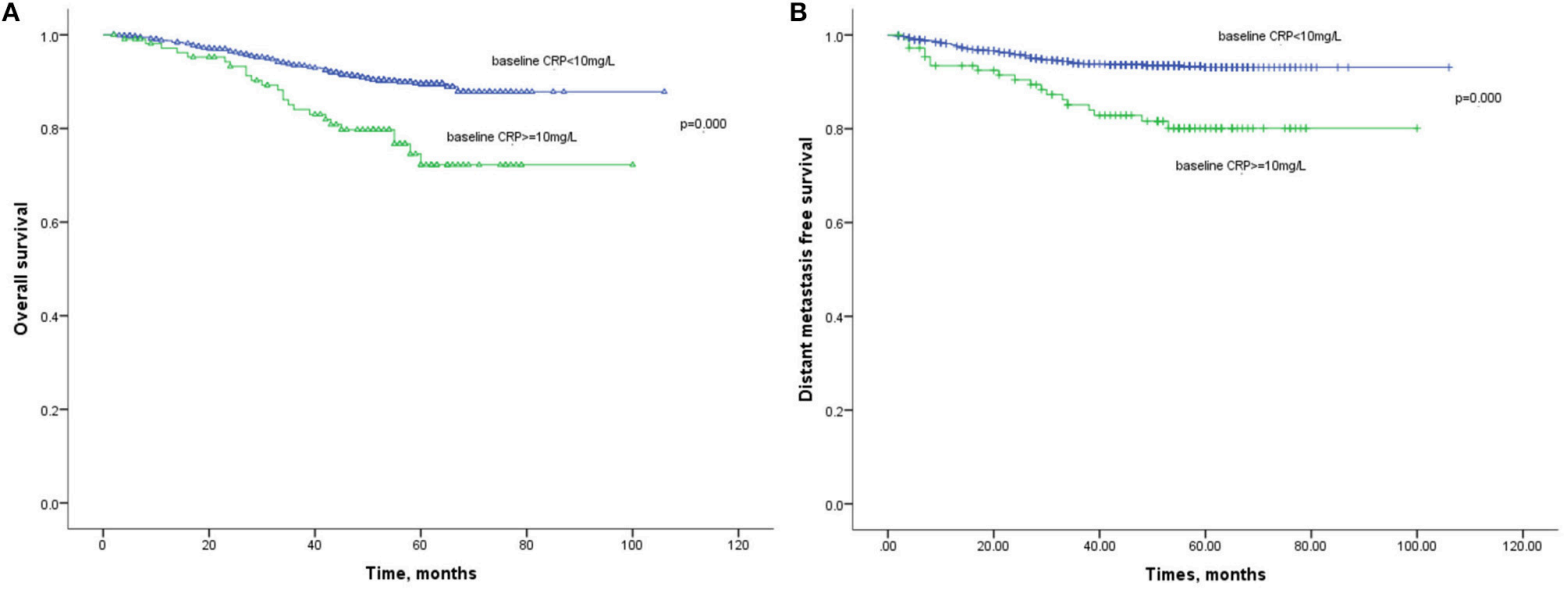
**(A)** Kaplan-Meier analysis of OS according to baseline CRP levels. **(B)** Kaplan-Meier analysis of DMFS according to baseline CRP levels.

The 1-year, 3-year, and 5-year OS rates in the normal baseline CRP group were 99, 91, and 88%, respectively (Figure 2A). Meanwhile, the 1-year, 3-year, and 5-year OS rates in the elevated baseline CRP group were 94, 80, and 71%, respectively, with a significant difference observed between the two groups (Table 2; HR: 2.541; 95%CI: 1.673–3.943; *p* < 0.001). Similarly, the 1-year, 3-year, and 5-year DMFS rates in the elevated baseline CRP group were markedly poorer than those in normal group (91 vs. 96%, 83 vs. 94%, and 79 vs. 93%; Figure 2B) respectively, suggesting a significant difference (HR: 3.001; 95%CI: 1.817–4.955; *p* < 0.001). Other risk factors, including advanced tumor stage, elevated CRP after treatment and CRP kinetics, were correlated with poor OS and DMFS rates (Table 2). By further, multivariate analysis (Table 3) was also performed, incorporating patient factors (age, gender, family history, smoking, hypertension, diabetes, chronic hepatitis B, and BMI), tumor factors (pathological type and TNM stage), treatment methods (radiotherapy and chemotherapy) and baseline CRP level as covariates. The baseline CRP level was confirmed as an independent prognostic factor for OS and DMFS (HR: 2.502, 95%CI: 1.510–4.148, *p* < 0.001; HR: 3.056, 95%CI: 1.751–5.333, *p* < 0.001), respectively.

**Table 2:** Univariate analyses of prognosis factors for OS and DMFS.

**Variables**	**OS**			**DMFS**		
	**HR**	**95% CI**	***p***	**HR**	**95% CI**	***p***
Age	2.187	1.557–3.071	0.000	1.322	0.897–1.950	0.159
Sex	1.875	1.22–2.881	0.04	1.746	1.050–2.903	0.032
Histology	0.969	0.541–1.737	0.917	1.044	0.501–2.175	0.909
BMI	0.624	0.437–0.890	0.009	0.704	0.464–1.070	0.100
Family history	0.903	0.624–1.305	0.586	0.792	0.504–1.244	0.311
Smoking	1.650	1.198–2.273	0.002	1.168	0.788–1.730	0.439
DM	1.176	0.435–3.177	0.749	0.434	0.061–3.113	0.407
Hypertension	1.502	0.833–2.709	0.177	0.866	0.352–2.126	0.753
Chronic HBV infection	0.599	0.361–0.993	0.047	0.683	0.381–1.225	0.201
Total stage	2.037	1.604–2.588	0.000	1.782	1.347–2.358	0.000
T stage	1.592	1.322–1.918	0.000	1.199	0.972–1.480	0.091
N stage	1.637	1.349–1.988	0.000	1.910	1.507–2.421	0.000
Radiotherapy	0.876	0.740–1.038	0.125	0.901	0.736–1.103	0.313
Chemotherapy	1.568	0.955–2.574	0.075	1.432	0.797–2.574	0.230
Baseline CRP	2.541	1.673–3.943	0.000	3.001	1.817–4.955	0.000
CRP during treatment	1.202	0.730–1.978	0.470	1.421	0.778–2.596	0.253
CRP after treatment	3.041	1.552–5.961	0.001	3.689	1.631–8.343	0.002
CRP change	2.610	1.592–4.279	0.000	2.816	1.496–5.302	0.001

**Table 3:** Multivariate analyses of prognosis factors for OS and DMFS.

**Endpoints**	**Baseline CRP**			**CRP after treatment**			**CRP kinetics**		
	**Variables**	**HR (95% CI)**	***p***	**Variables**	**HR (95% CI)**	***p***	**Variables**	**HR (95% CI)**	***p***
OS	Sex	0.447(0.254–0.785)	0.005	Sex	0.310(0.113–0.847)	0.022	Sex	0.184(0.053–0.642)	0.008
	Age	2.080(1.385–3.125)	0.000	Age	2.152(1.128–4.104)	0.020			
	BMI	0.656(0.433–0.994)	0.047	BMI	0.443(0.234–0.840)	0.013			
	TNM	1.663(1.234–2.239)	0.001	TNM	1.978(1.219–3.210)	0.006	TNM	1.655(1.101–2.805)	0.043
	Baseline CRP	2.502(1.510–4.148)	0.000	CRP after treatment	2.892(1.334–6.271)	0.007	CRP kinetics	2.512(1.452–4.346)	0.001
DMFS	Sex	0.423(0.227–0.785)	0.006	Sex	0.120(0.027–0.543)	0.006	Sex	0.066(0.008–0.517)	0.010
	TNM	1.696(1.193–2.411)	0.003						
	Baseline CRP	3.056(1.751–5.333)	0.000	CRP after treatment	4.876(2.008–11.836)	0.000	CRP kinetics	3.389(1.734–6.625)	0.000

**Figure 3:**
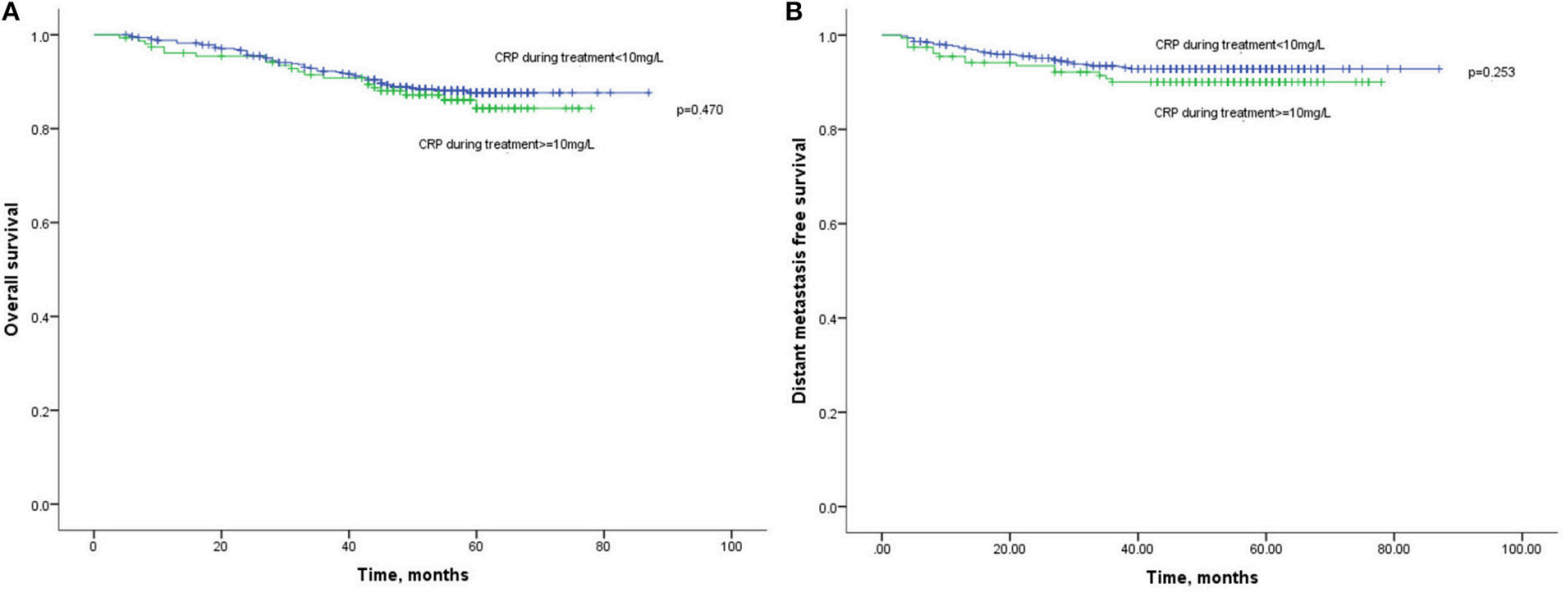
**(A)** Kaplan-Meier analysis of OS according to CRP levels during treatment. **(B)** Kaplan-Meier analysis of DMFS according to CRP levels during treatment.

Besides, the OS (Figure 3A) and DMFS (Figure 3B) curves were compared between patients with elevated and non-elevated CRP level during treatment, with no significant differences found. Further, univariate analysis also suggested that CRP level during treatment was insignificant with OS (HR: 1.202; 95%CI: 0.730–1.978; *p* = 0.470) or DMFS (HR:1.421, 95%CI: 0.778–2.596; *p* = 0.253). All together confirmed that CRP level during treatment was not a prognostic factor for the current cohort (Table 2).

**Figure 4:**
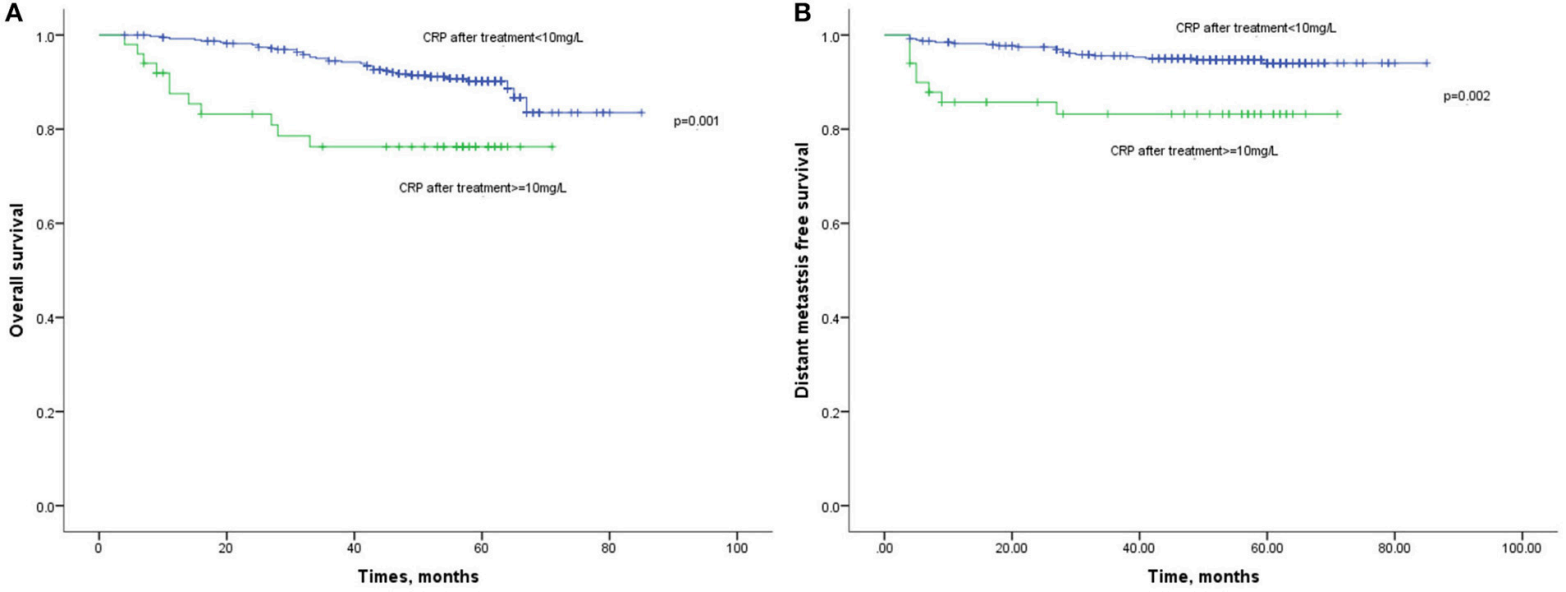
**(A)** Kaplan-Meier analysis of OS according to CRP levels after treatment. **(B)** Kaplan-Meier analysis of DMFS according to CRP levels after treatment.

Lastly, the 1-year, 3-year, and 5-year OS rates in elevated and normal CRP levels after treatment were 83 and 98%, 76 and 92%, and 76 and 87%, respectively (Figure 4). It suggested that the elevated CRP after treatment was associated with a poor survival (HR:3.041, 95%CI: 1.552–5.961, *p* = 0.001). Similarly, elevated CRP after treatment also indicated significantly decreased DMFS compared with normal CRP after treatment (HR: 3.689, 95%CI: 1.631–8.343, *p* = 0.002). Multivariate analysis revealed CRP level after treatment was an independent prognostic factor of OS (HR: 2.892, 95%CI: 1.334–6.271, *p* = 0.007) and DMFS (HR: 4.876, 95%CI: 2.008–11.836, *p* < 0.001).

### CRP Kinetics and Survival

**Figure 5:**
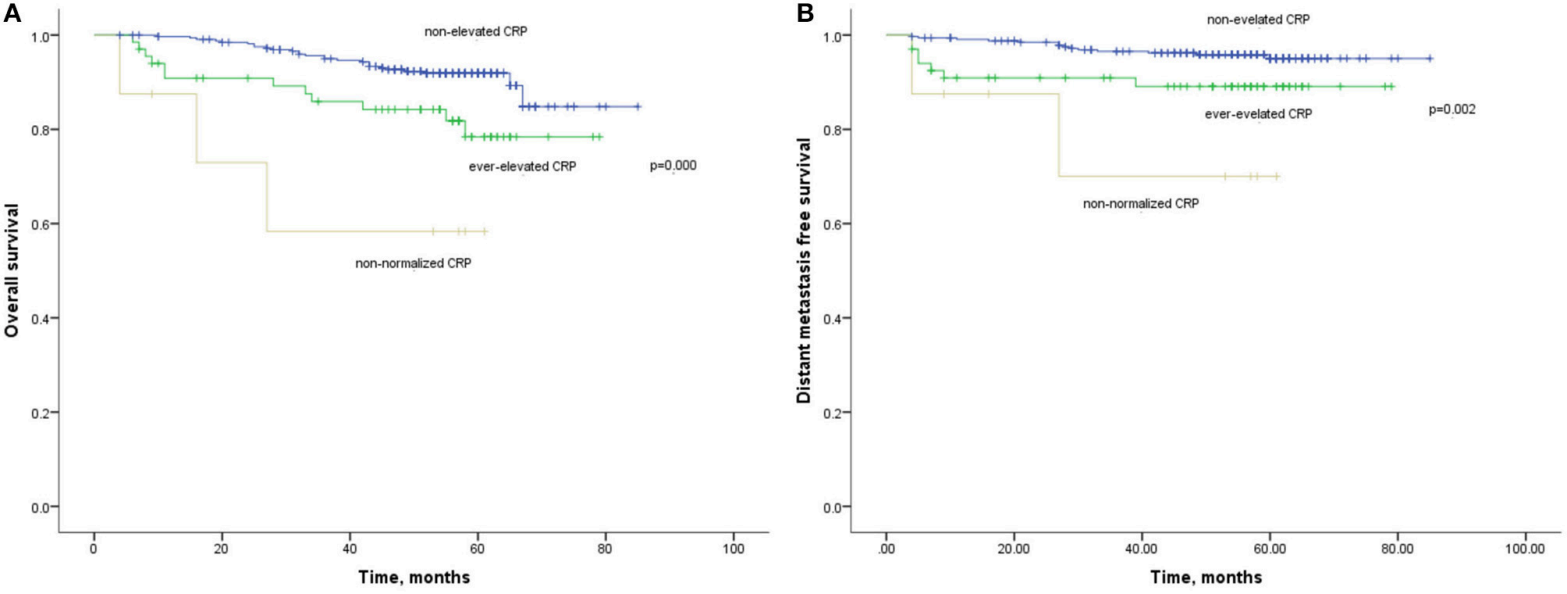
**(A)** Kaplan-Meier analysis of OS according to CRP kinetics. **(B)** Kaplan-Meier analysis of DMFS according to CRP kinetics.

The 1-year, 3-year, and 5-year OS rates for patients with non-elevated CRP was 98, 93, and 89%, which were significantly improved compared with those with ever-elevated (91, 84, and 79%) and non-normalized CRP (72, 58, and 58%; Figure 5). As for the DMFS rates, the non-elevated CRP group had 1, 3, 5-year rates of 98, 96, and 94%, respectively. The ever-elevated group and non-normalized group had 1, 3, 5-year rates of 91 and 87%, 89 and 69%, and 97 and 69%, respectively. There were significantly differences in OS (HR:2.610, 95%CI: 1.592–4.279, *p* < 0.001) and DMFS (HR:2.816, 95%CI: 1.486–5.302, *p* = 0.001) among three groups. Multivariate analysis demonstrated that CRP kinetics was an independent prognostic factor of OS (HR:2.512, 95%CI: 1.452–4.346, *p* = 0.001) and DMFS (HR:3.389, 95%CI: 1.734–6.625, *p* = 0.001).

## Discussion

In the present study, we found that pre-treatment and post-treatment levels of CRP, rather than CRP during treatment, were helpful to predict prognosis of non-metastatic NPC patients. Furthermore, we assessed the effect of CRP kinetics during treatment and found that CRP kinetics was an independent prognostic factor for prognosis of NPC patients. To the best of our knowledge, this study was the first report to confirm the prognostic significance of pre-treatment CRP, post-treatment CRP, and CRP kinetics during treatment for non-metastatic NPC patients.

Inflammation is known as the seventh hallmark of tumor formation and development, which is recognized to promote the initiation, progression and metastasis of cancer (24–27). CRP, as an acute-phase protein, is a non-specific protein reacting to acute inflammation, infection, and tissue damage (13). It has been regarded as a valuable inflammatory marker in cardiovascular disease (28), diabetes (29), and chronic hepatitis B (30, 31). Recently, further researches have demonstrated a correlation of high CRP level with a poor prognosis in colorectal cancer (15), osteosarcoma (32), hepatocellular carcinoma (16), and other cancers, as well as in NPC patients (19, 21). The current study has demonstrated pre-treatment CRP and post-treatment CRP are both independent and significant prognostic indicators of non-metastatic NPC patients, and the finding is consistent with previous studies.

We further analyzed the relationship of the CRP level in different treatment phases with prognosis, which was relatively lacking in previous studies. Interestingly, CRP level during treatment showed no relationship with OS and DMFS in NPC patients. As we know, acute radiotherapy-induced mucosal reaction, bacterial and fungal infections, chemotherapy-induced hepatic injury, renal impairment, and cardiac damage commonly happened during the whole process of radical radiotherapy and induction and concurrent chemotherapy. The elevation of CRP during treatment may be due to the inflammation, infection and tissue damage mentioned above, therefore the effect of predicting prognosis is covered over.

Recently, research of relationship between tumor biomarker kinetics and patient prognosis has been attracting more attention. As an EBV-related malignance, EBV DNA has been used in diagnosis, predicting prognosis, and monitoring in NPC patients (8, 33, 34). However, there is still no unified detection method in circulating EBV DNA quantification. CRP kinetics has also been revealed as a prognostic indicator in metastatic renal cell carcinoma treated with TKIs (35). Xia et al. reported that baseline CRP level and CRP kinetics may be useful to predict prognosis of metastatic NPC patients treated with palliative chemotherapy (22). To date, there is no related research revealing the relationship with CRP and prognosis in non-metastatic NPC patients treated with radical radiotherapy. Our current study has demonstrated that CRP kinetics is an independent prognostic indicator in non-metastatic NPC patients. The prognosis of patients with non-elevated CRP during radical treatment is better than patients with ever-elevated and non-normalized CRP. Since CRP is more regular carried out in basic hospitals in China, the CRP monitoring may be an effective and economical in basic hospitals in China during follow-up.

This study had several limitations. First, this was a retrospective, single-center analysis and patient-selection bias was inescapable. Second, EBV DNA test was not regularly performed in this cohort due to the limitation of times. Therefore, the interaction of EBV DNA expression and CRP level was not explored in the current study. At last, biological mechanisms that unravel the prognostic value of CRP level and kinetics in NPC were not revealed.

In conlusion, the pretreatment CRP level, CRP after treatment, and CRP kinetics may be a useful prognostic indicator in non-metastatic NPC patient treated with radical radiotherapy. Further research including large prospective studies is required to draw more definitive conclusions.

## Ethics Statement

This study was carried out in accordance with the recommendations of Institutional Review Board of First Affiliated Hospital of Sun Yat-sen University with written informed consent from all subjects. All subjects gave written informed consent in accordance with the Declaration of Helsinki. The protocol was approved by the First Affiliated Hospital of Sun Yat-sen University.

## Author Contributions

RC and YZ carried out the search strategy and drafted the manuscript. YY participated in the data extraction and statistical analysis. QZ and SH participated in literature search and data extraction. YC and YR conceived of the study, and participated in its design and coordination, and helped to draft the manuscript. All authors read and approved the final manuscript.

### Conflict of Interest Statement

The authors declare that the research was conducted in the absence of any commercial or financial relationships that could be construed as a potential conflict of interest.

## Abbreviations

CRP: C-reactive protein
NPC: nasopharyngeal carcinoma
OS: overall survival
DMFS: distant metastasis free survival
EBV: Epstein-Barr virus
MRI: magnetic resonance imaging
FDG: PET/CT fluorodeoxyglucose positron emission tomography-computed tomography
UICC/AJCC: International Union against Cancer/American Joint Committee on Cancer
3D-CRT: 3-dimensional conformal radiation therapy
IMRT: Intensity Modulated Radiation Therapy.
